# Tiller estimation method using deep neural networks

**DOI:** 10.3389/fpls.2022.1016507

**Published:** 2023-01-13

**Authors:** Rikuya Kinose, Yuzuko Utsumi, Masakazu Iwamura, Koichi Kise

**Affiliations:** ^1^ Graduate School of Engineering, Osaka Prefecture University, Sakai, Japan; ^2^ Graduate School of Informatics, Osaka Metropolitan University, Sakai, Japan

**Keywords:** tiller number estimation, deep neural network (DNN), pretext task, self-supervised learning, regression

## Abstract

This paper describes a method based on a deep neural network (DNN) for estimating the number of tillers on a plant. A tiller is a branch on a grass plant, and the number of tillers is one of the most important determinants of yield. Traditionally, the tiller number is usually counted by hand, and so an automated approach is necessary for high-throughput phenotyping. Conventional methods use heuristic features to estimate the tiller number. Based on the successful application of DNNs in the field of computer vision, the use of DNN-based features instead of heuristic features is expected to improve the estimation accuracy. However, as DNNs generally require large volumes of data for training, it is difficult to apply them to estimation problems for which large training datasets are unavailable. In this paper, we use two strategies to overcome the problem of insufficient training data: the use of a pretrained DNN model and the use of pretext tasks for learning the feature representation. We extract features using the resulting DNNs and estimate the tiller numbers through a regression technique. We conducted experiments using side-view whole plant images taken with plan backgroud. The experimental results show that the proposed methods using a pretrained model and specific pretext tasks achieve better performance than the conventional method.

## 1 Introduction

A tiller is a branch of a grass plant. For grain bearing members of the grass family, the number of fertile shoots per unit area, number of grains per ear, and size of grains are the determinants of yield. Therefore tillering is one of the traits targeted for phenotyping, particularly as tiller number can vary through the life of a plant in response to environmental and genetic factors (Xie et al., 2015). Therefore, it is one of the traits that is targeted for phenotyping. Destructive surveys have commonly been used to count the number of tillers, because they are hard to count visually; leaves and tillers look similar, and the density of tillers tends to be highest at the base of the plant. However, destructive surveys present a bottleneck to phenotyping tasks because they are time-consuming and labor-intensive, making it impossible to trace the growth of the plants. To achieve nondestructive and automatic tiller number estimation, several image-based methods have been proposed (Fahlgren et al., 2015; Boyle et al., 2016).

However, the estimation accuracy of the conventional image-based methods is generally poor. However, the estimation accuracy of the conventional image-based methods is generally poor. To estimate the tiller number, image-based approaches use hand-crafted features^1^ such as the area and aspect ratio of a plant within an image and the output of the Frangi filter (Frangi et al., 1998) for linear regression. As these methods only use a few heuristic features of the plants’ appearance, they do not take full advantage of the information contained in the images. The recent development of image recognition techniques using features learned by deep neural networks (DNNs) surpasses the performance of conventional hand-crafted feature-based methods (Taigman et al., 2014; Simonyan and Zisserman, 2015; Schroff et al., 2015; Hu et al., 2018). DNNs learn image features directly from the image appearance. Thus, the features learned by DNNs take full advantage of the plants’ appearance. This motivates us to use DNNs to learn features as a means of realizing high-accuracy tiller number estimation.

DNNs requires large volumes of training data, consisting of pairs of an image and the corresponding ground truth. The image dataset of Setaria plants (Gehan et al., 2015) contains only around 600 images with the corresponding tiller numbers because the operation of counting the tiller numbers is time-consuming and labor-intensive, as mentioned above. Therefore, it is difficult to prepare sufficient training data for DNNs, making it almost impossible to apply DNN-based methods for tiller number estimation.

As a lack of training data is commonly encountered in the field of computer vision and pattern recognition, several methods have been developed to enable DNNs to be used with small-scale data. For example, transfer learning (Huang et al., 2019) transfers the network learning to another dataset, semi-supervised learning (Miyato et al., 2019) uses partly labeled data for learning, and self-supervised learning (Gidaris et al., 2018) uses self-generating labels. Some self-supervised learning methods that learn features by solving other tasks have achieved comparable performance to supervised methods (Noroozi et al., 2017; Gidaris et al., 2018; Noroozi et al., 2018). These other tasks are called “pretext tasks,” and they can be applied to problems in which large numbers of unlabeled data are available.

In this paper, we describe the use of self-supervised learning and transfer learning to estimate the tiller number, even though there are relatively few training data (Utsumi et al., 2019; Kinose et al., 2022). To the best of our knowledge, this is the first attempt to use deep learning-based image features in nondestructive tiller number estimation using a single RGB image. We apply transfer learning to the estimation task and examine how the features learned from other data affect the estimation. We also set some pretext tasks for learning DNNs and evaluate how the pretext tasks enhance the estimation performance. Experimental results show that the proposed method outperforms the conventional method and that the pretext tasks enhance the estimation accuracy. The results showed that when using the framework of the proposed method, the plant trait can be estimated accurately using deep-learning even though few training data are acquired.

### 1.1 Related work

#### 1.1.1 Tiller number estimation

DNN-based tiller number estimation techniques have already been proposed (Deng et al., 2020; Wu et al., 2021). Deng et al. (Deng et al., 2020) applied DNN-based image detection to stubble images as a means of counting the tillers. However, this method requires a destructive survey, making it difficult to track the growth traits of the plants. The idea of counting tillers proposed by Wu et al. (Wu et al., 2021) is almost the same as that developed by Deng et al., except that the images are obtained using micro-CT. Unfortunately, micro-CT is too expensive to be widely used. Different from these methods, the proposed requires only an RGB image to estimate the tiller numbers. Therefore, it is suitable for easy and high-throughput phenotyping.

#### 1.1.2 Image-based plant phenotyping using DNNs

The most common task for DNN-based individual phenotyping is leaf counting because an image dataset of Arabidopsis thaliana was released (Minervini et al., 2016). The dataset has since been used in the development of many methods (Aich and Stavness, 2017; Ubbens et al., 2018; Ward et al., 2018). However, the dataset has few image data in which the number of leaves is identified. Therefore, techniques that artificially increase the number of data using data synthesis based on plant models have been proposed, enabling DNNs to be applied to small sets of labeled data (Ubbens et al., 2018; Ward et al., 2018). This data synthesis approach cannot be easily applied to tiller number estimation because the structure of grass plants is too complicated to model.

In addition to counting the leaves of Arabidopsis thaliana, many traits have been estimated using DNNs. Roots are another typical subject for trait estimation using DNN-based image analysis. For example, segmentation algorithms for root regions (Han and Kuo, 2018; Wang et al., 2019; Gaggion et al., 2021) and root structure analysis based on the characterization of roots (Wu et al., 2018; Yasrab et al., 2019) have been proposed. Certain traits of wheat, which is a member of the grass plant family, have also been estimated, such as the number of spikes and spikelets (Pound et al., 2017) and the emergence and biomass (Aich et al., 2018).

#### 1.1.3 Pretext tasks

Various pretext tasks have been proposed. For example, colorizing images (Zhang et al., 2016), solving jigsaw puzzles (Noroozi and Favaro, 2016), predicting image rotations (Gidaris et al., 2018), and counting the number of objects within an image (Noroozi et al., 2017) have been used for representation learning. The learned representations are used for image segmentation, image recognition, and object recognition.

In establishing the proposed method, we set some pretext tasks for tiller number estimation according to these previous methods. The application of pretext task means that tiller number estimation can be conducted using DNNs, even if few labeled data are available.

## 2 Materials and methods

We explain how the proposed method estimates the tiller number from an image. We adopt regression-based estimation for tiller counting, as in conventional image-based tiller number estimation methods (Fahlgren et al., 2015; Boyle et al., 2016). This is because regression-based estimation is more practical than the detection-based method. For examples, in the leaf counting task of Arabidopsis thaliana, regression-based method show better accuracy than the object-detection-based method Ubbens and Stavness (2017), and many regression-based method have been proposed Giuffrida et al. (2015); Dobrescu et al. (2017); Aich and Stavness (2017). Tillers have a similar appearance to leaves, and so it is hard to detect tillers from images. Moreover, the tillers become too dense to detect as the plant grows. Therefore, we adopt a regression-based method.

**Figure 1:**
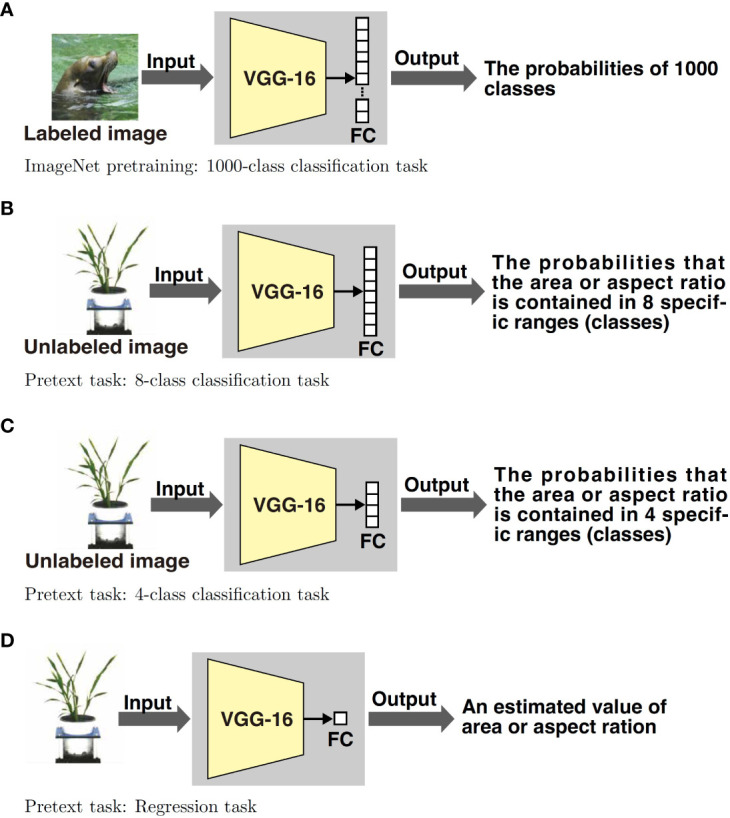
We use four feature training methods. **Figure 1A** shows a typical supervised training method, which uses the ImageNet dataset. The dataset consists of 1000 classes; thus the output layer (the last fully connected (FC) layer) of the network consists of 1000 elements. The DNN model is trained by updating the parameters in order to reduce the error between the output of the DNN and the ground truth. We use a pretraining model available on the web. **Figures 1B–D** show self-supervised methods using pretext tasks; they use 8-class classification, 4-class classification, and regression tasks. Hence, their last layers consist of eight, four, and one element, respectively. The pretext tasks in the proposed method estimate the area or aspect ratio of the plant within the input image. The images for training are unlabeled with the tiller numbers. The ground truth of the area and aspect ratio of the plant within the image are calculated by using image processing beforehand.

**Figure 2:**
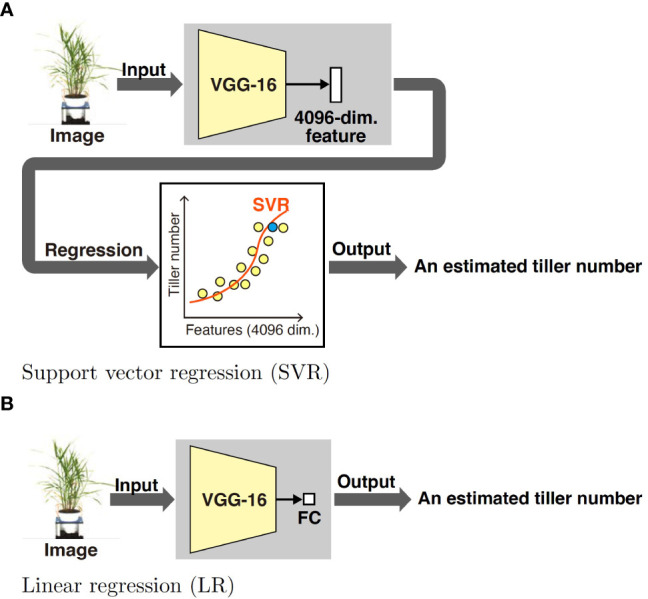
We propose two tiller number estimation methods using different regression models: support vector regression (SVR) and linear regression (LR). Both methods use the pretrained DNN model in **Figure 1**; more specifically, we use the pretrained the VGG-16 model. In both methods, a plant image taken from the side is used for estimating the number of tillers. In SVR, a 4096-dimensional feature is extracted from the input image using the pretrained DNN. The parameters of SVR are estimated using features extracted from labeled images; the features and tiller numbers are used as independent and dependent values, respectively. In LR, a new FC layer consisting of an element is prepared. Using labeled images, the DNN is trained. Then, the DNN outputs an estimated tiller number for an input unlabeled data.

The proposed method consists of two parts: feature learning and estimating the tiller numbers. **Figure 1** shows an overview of the feature learning part. The VGG-16, which is one of the most popular DNN models, is pretrained on the ImageNet classification task and pretext tasks. After pretraining, fully connected layers are discarded and new ones are prepared according to the tasks. Although it is common to use the ImageNet dataset for pretraining (**Figure 1A**), we use images without tiller number labels on the pretext tasks (**Figures 1B–D**). The labels on the pretext tasks were acquired automatically by image processing. **Figure 2** shows an overview of the tiller number estimation part. In the tiller number estimation, the features are extracted by the trained networks, which purged the FC layers. The dimensionality of the feature is 4096. The tiller numbers are estimated by regression; the features tiller numbers are used as an independent and dependent value, respectively. A small number of images with tiller numbers are used for training the regression model of tiller number estimation. Image resources and processing, the pretrained model, pretext tasks, and regression models are now described in detail.

### 2.1 Image resources and processing

We used the dataset that appears in Gehan2015^2^ ^,^ ^3^ The first row in **Figure S1** shows some examples of the dataset. The dataset contains 25,570 images of potted Setaria taken from the side in a controlled laboratory environment. The species of the Setaria are S. viridis (A10), S. italica (B10), and eight RILs (RIL020, RIL070, RIL098, RIL102, RIL128, RIL133, RIL161, RIL187) from an S. viridis × S. italica population. We used side-view whole plant images in the dataset. The images are in RGB color, and the image resolution is 2,454 × 2, 056 pixels. In the dataset, 576 images have tiller numbers that were counted manually. Thus, there are 24,994 unlabeled images that have no tiller number. Many of the unlabeled images were taken at the same time as the labeled images. To avoid mixing unlabeled data that are similar to the labeled data, we only used the 22,110 unlabeled images that were not taken at the same time as the labeled images. There are some images in which plants stick out from the frame in the dataset.

We normalized the images before the experiments. The magnification of the images was artificially determined according to the plant growth degree. As the first row of **Figure S1** shows, the pot size and the background differ depending on the plant size. If such images were used for learning, the network may learn features that focus on changes in the pots and backgrounds. To avoid the network focusing on parts unrelated to the plants, we normalized the images. We executed the normalization in a semi-automatic manner: We sampled an image from each magnification and manually cropped a rectangle area that included the whole plant area to remove the background. Because all plants were in pots of the same size, the images were resized so that the pot size was the same. After removing the background, we manually determinated the upper part of the pot in the cropped image. The cropped image was translated to place the center of the pot in the center of the image and resized the cropped image because the pot size was 32 pixels. The images were padded with white pixels to make the images square for input to the network. Finally, the size of the image is 224 × 224 pixels. The rest of the images were automatically cropped in the same area, translated by the same amount, and scaled to the same size as the sample image. We confirmed that all plant areas were not cut off in the normalized images. All procedures were performed using OpenCV, and we used the bicubic method for pixel interpolation when the images were resized. There were no images that some parts of larger plants are out of the frame caused by the normalization.

We used the 22,110 unlabeled images for the training of the pretext tasks and the 576 images which has tiller numbers for evaluating tiller number estimation. The unlabeled images did not overlap with the labeled 576 images.

### 2.2 Feature extraction

#### 2.2.1 Pretrained model

A sufficient number of labeled data for training is required for a DNN to achieve good performance. However, it is often the case that a sufficient number of labeled data are not available. A typical solution is to use a pretrained model. Usually, a pretrained model is trained on a large dataset, such as the ImageNet dataset (Deng et al., 2009), in a classification task. The use of the pretrained model is considered reasonable from the observation that a DNN trained on a large dataset in a task extracts effective features in a different task.

There are some standard DNNs used in the field of computer vision. One of such DNNs is the VGG model (Simonyan and Zisserman, 2015). Compared to ResNet He et al. (2016), another standard DNN, GG is simple and easy to train. Although ResNet often achieves better performance in a complex task with a lot of labeled data for training, VGG often performs equivalently in a simple task with less labeled data. Since our task is simple, we use the 16-layer VGG model, which is called VGG-16.

#### 2.2.2 Pretext tasks

As mentioned in Section 1, it is redimpractical to learn the feature expression from the tiller number estimation task directly because of the shortage of labeled training data. Thus, we use pretext tasks to learn the feature expression, and estimate the tiller number using the learned features.

The VGG-16 model (Simonyan and Zisserman, 2015) is trained using pretext tasks that predict appearance related values acquired automatically from a plant image. As shown in **Figure 1**, we set two pretext tasks: estimating the area of a plant within an image and estimating the aspect ratio of a plant. We consider the area and aspect ratio because they were used as the dependent variables for estimating the tiller numbers in a previous study (Fahlgren et al., 2015) and are expected to provide good feature expressions for tiller number estimation.

We investigate two methods of estimating the area or aspect ratio of the plant within an image in the pretext tasks: the area or aspect ratio themselves and the discretized area or aspect ratio. When estimating the area or aspect ratio itself, as shown in **Figure 1D**, the network is trained so that the output is the area or aspect ratio. We call the pretext task that estimates the value itself “regression task,” because in this case, the pretext task can be regarded as a regression task with the image as the independent value and the continuous values of area and aspect ratio as the dependent values. In the case of estimating discrete area or aspect ratio, instead of outputting a numerical area or aspect ratio, the network predicts the discretized area or aspect ratio of the plants in the input image, as shown in **Figures 1B** and **C**. Therefore, predicting discrete area or aspect ratio is equivalent to classification. We call the pretext task estimating the discretized values “classification task.”

We conducted network training on the pretext tasks using the normalized images. The ground truth of the pretext tasks was calculated automatically using image processing. Following the “Single plant RGB image workflow” in the PlantCV tutorial^4^, the normalized images were translated into HSV and Lab images, and thresholding was applied to the saturation component of the HSV images and the *a* and *b* components of the Lab images. The plant area was then segmented by taking the logical sum of the threshold results. The area and aspect ratio were calculated from the segmented plant area. All processes were conducted using PlantCV ^5^. The images were divided into four or eight classes in the classification task according to the area and aspect ratio values, respectively. The images were divided so that the number of images in each class became the same.

The network was trained to predict the class to which the input image belongs. In the regression task, the network was trained to predict the area or aspect ratio of the input images. Both tasks used 80% of the images for training and 20% of the images for testing. The network used for training was the VGG-16 model pretrained by the ImageNet dataset. The mini-batch size, learning rate, and epochs for the training were set as 128, 0.0001, and 200, respectively. We applied horizontal and vertical flip data augmentation. We trained the network 12 times with the above condition and adopted the model that gave the lowest training error for tiller number estimation. We used the Keras TensorFlow2 backend to execute the training process.

### 2.3 Tiller number estimation

We use two regression models to estimate the tiller numbers, namely support vector regression (SVR) and linear regression (LR).

SVR involves the application of a support vector machine to regression. The most significant advantage of SVR is that it deals with nonlinear regression problems through the same framework as linear SVR. In SVR, a feature space can be mapped to a space of much higher dimension using a kernel function. When the kernel function is nonlinear, SVR can deal with nonlinear regression problems. Moreover, SVR can learn from small-sized datasets. Hence, we apply SVR to tiller estimation. Specifically, we extract features from labeled images using the models described in Sections 2.2.1 and 2.2.2, and then apply SVR.

We also use linear regression (LR) for the estimation task. LR is one of the simplest regression methods and is equivalent to a fully connected neural network without a hidden layer. Because it is easy to implement LR with methods using DNN-based features, we apply LR for the estimation task. As with SVR, we learn the LR model using the features extracted from labeled images.

We estimated the tiller number using the features extracted by pretext-task-trained and ImageNet pretrained models. In the case of SVR, we used scikit-learn^6^ for the implementation, which is one of the most popular machine learning libraries for Python. The radial basis function was used as the kernel. The cost parameter *C* and parameter ϵ were set to 100 and 1.0, respectively, and default values were used for the other parameters. LR was implemented by adding two fully connected layers to the VGG-16 model. We then trained only the added layer while freezing VGG-16.

## 3 Results

### 3.1 Tiller number estimation

We executed the proposed tiller number estimation using features extracted by models trained by pretext tasks and the pretrained model to reveal the difference between the feature extraction models. We used six-fold cross-validation to calculate the accuracy of the tiller number estimation. That is, the images were divided into six groups and the regression models were trained with five groups and validated with the remaining group. This process was repeated until all groups had been used for validation. The accuracy of the model was calculated by taking the average of each of the six cross-validation tasks. We adopt the mean absolute error (MAE) to evaluate the accuracy of the proposed method. We used the GPU, NVIDIA TITAN RTX, for training the network with the pretext tasks, and the CPU server, which has Opteron 6348 CPU (2.8GHz) and 512GB memory for estimating the tiller numbers. We also executed the conventional method proposed in Fahlgren2015 on the same CPU server.

For fare comparison, we executed the method proposed by Fahlgren et al., 2015 with the dataset we used. Fahlgren et al., 2015 estimated plant fresh weight using plant area on an original image by the following equation.


(1) $$M_{fw}=3.755\times 10^{-5}A_{sv}-0.2704$$

*M_fw_*, *A_sv_* are estimated plant fresh weight and area of the plant in an image, respectively. Then, the tiller number was estimated by using the estimated fresh weight and aspect ratio of the plant in the image as follows:


(2) $$TC=0.22M_{fw}-2.19HW+5.26,$$

*TC*,*HW* are the tiller number and aspect ratio of the plant in the image. We cannot directly apply the equations as we resized the original images. Therefore, we estimated the parameters in eq 1 using images with fresh weight. The original dataset we used in this paper(https://figshare.com/articles/dataset/DDPSC_Phenotyping_Manuscript_1_Files/1272859) has 158 images that have fresh weight. We normalized the images in the same manner as other images used for the experiments and estimated the parameter of eq. 1. We estimated the parameter 2, and evaluated the accuracy of the equation with the same 576 images, which have tiller numbers, as the proposed methods were evaluated using six-fold cross-validation. The estimated parameters of eq. 1 are as follows:


(3) $$M_{fw}=0.005790A_{sv}-0.3372.$$

The coefficient of determination of eq. 3 *R* ^2^ was 0.9589. The estimated parameters of eq. 2 are as follows:


(4) $$TC=0.1786M_{fw}-1.1102HW+3.885$$

**Figure 3:**
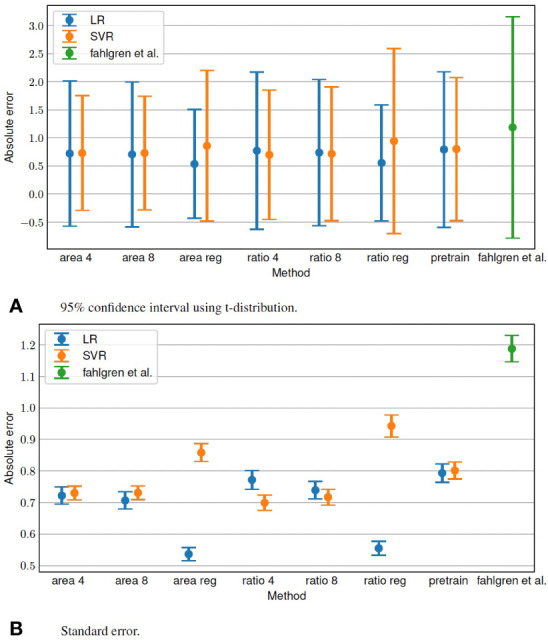
Standard error and 95% confidence interval of tiller number estimation.

**Table 1** presents the MAE when using SVR and LR to estimate the tiller numbers. The total running time for estimation per image was 98, 191, and 0.6 ms when using LR with the proposed method, SVR with the proposed method, and (Fahlgren et al., 2015), respectively. We also evaluated the standard error and 95% confidence interval of the estimation, as shown in **Figure 3**.

**Table 1:** MAE of estimation results when using SVR and LR.

		Pretext task					
		Area			Aspect ratio		
Reg. model	Pretrained	4 cls.	8 cls.	Reg.	4 cls.	8 cls.	Reg.
(r)1-1(r)2-8 SVR	0.80	0.74	0.78	0.91	0.73	0.73	1.00
LR	0.79	0.74	0.71	0.57	0.96	1.06	0.62

“Reg. model” stands for the regression model used for tiller number estimation. “Reg.”, “4 cls.”, “8 cls.” in the pretext task stands for Regression task, 4 class classification task, and 8 class classification task, respectively.

### 3.2 Individual estimation results

**Figure 4:**
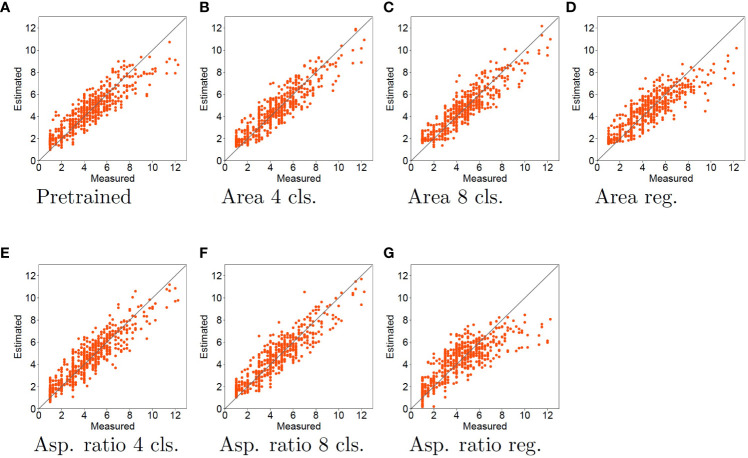
Experimental results using SVR for tiller number estimation. Horizontal and vertical axes represent the measured and estimated tiller numbers, respectively. Each red dot denotes a sample for the estimated tiller number. The black line indicates the case where the measured and estimated data match. Therefore, the closer the points are to the black line, the more accurate is the estimation.

**Figure 5:**
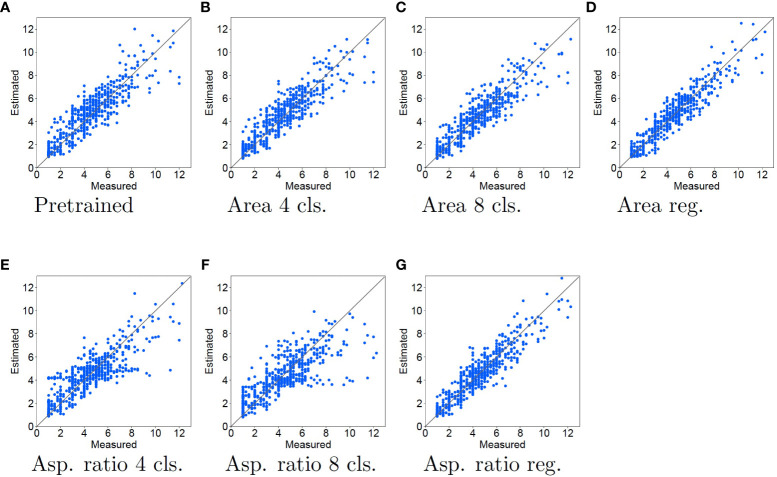
Experimental results using LR for tiller number estimation. The contents of the graph are the same as in **Figure 4**.

The measured tiller number (horizontal axis) and estimated tiller number (vertical axis) are compared in **Figures 4**, **5** for the cases using SVR and LR for tiller number estimation, respectively.

## 4 Discussion

This proposed method is the first attempt to apply self-supervised learning using pretext tasks for plant phenotyping, as far as we know. Plant datasets have insufficient labeled data for applying DNNs. The proposed semi-supervised method for estimating the number of tillers requires only a few labeled data. Therefore, the proposed method show good estimation accuracy. The best MAE of 0.57 is achieved when the area regression is used for the pretext task and LR is used to predict the tiller numbers. The MAE by Fahlgren et al. (Fahlgren et al., 2015) was 1.187. Note that it is not possible to make a general comparison because of the different image usage conditions and because Fahlgren et al. (Fahlgren et al., 2015) used a different number of images to that in the dataset (Gehan et al., 2015). However, it appears that the proposed method achieves good accuracy. The proposed method show good estimation accuracy. The best MAE of 0.57 is achieved when the area regression is used for the pretext task and LR is used to predict the tiller numbers. The MAE by Fahlgren et al. (Fahlgren et al., 2015) was 1.187. Note that it is not possible to make a general comparison because of the different image usage conditions and because Fahlgren et al. (Fahlgren et al., 2015) used a different number of images to that in the dataset (Gehan et al., 2015). However, it appears that the proposed method achieves good accuracy.

To clarify the effect of feature learning in the pretext tasks, we compared the accuracy of the pretext tasks and pretrained models. Many of the pretext tasks resulted in higher accuracy than using the pretrained model. Therefore, learning features using pretext tasks contributes to improving the accuracy of estimating tiller numbers.

The tiller number estimation accuracy depends on the trait estimated in the pretext task. The tiller number estimation accuracy is better when the area is used in the pretext task than when the aspect ratio is used. Therefore, the features learned in the pretext task using the area are more effective for tiller estimation than those learned from the aspect ratio.

The pretext task that gives the better tiller number estimation accuracy also depends on the tiller number estimation method. When SVR is used, the application of classification in the pretext task results in better accuracy than regression. In contrast, when LR is used, the application of regression in the pretext task achieves better accuracy than classification.

There is clearly a different tendency when SVR and LR are used for tiller number estimation. As shown in **Figure 4A**, when the pretrained model features are used with SVR, the estimated tiller number is substantially underestimated when the measured tiller number is high. In **Figures 4B, C, E, F**, orange dots are distributed close to the diagonal lines. This means that the accuracy of the tiller number estimation improves for samples with larger tiller numbers when the features learned by classification tasks are used, compared with pretrained model features. Thus, using classification for the pretext tasks improves the tiller number estimation accuracy. However, when using regression for the pretext tasks, the estimation accuracies are worse than those with the pretrained model. In particular, as shown in **Figures 4D, G**, orange dots are plotted in the rightmost of the figures [approximately 9 to 12 of measured (horizontal axis)],. This means that the estimation results for samples with larger tiller numbers are worse than those using the pretrained model.

When regression and LR were used for the pretext task and tiller number estimation, respectively, dots are close to the diagonal line, as shown in **Figures 5D, G**. This means that the estimation accuracy improves for all samples. In particular, comparing the pretrained model with the regression pretext tasks, the top right dots of regression pretext tasks is more close to the diagonal lines. This means that the accuracy is enhanced for samples with large measured tiller numbers. In contrast, as shown in **Figures 5B, C, E, F**, dots are vary widely from the diagonal lines. This means that when the features learned by the classification task are used, the estimation accuracy is the same or worse than that of the pretrained model. When the aspect ratio is used for the classification task, the estimation accuracy becomes worse, with the estimated tiller numbers consistently lower than the measured values.

We used images which are taken well-controlled lab environment and taken separately. Therefore, the proposed method would work well on images taken in a similar environment but not on images taken in a different environment. For example, if the images were taken in the field, multiple plants would appear in the images. In this case, we need to recognize the individual plants and apply the proposed method to each plant. However, when the plans are crowded, recognizing individual plants in side-view images is difficult for current image recognition. Thus, the proposed method is hard to apply to the images taken in the field.

However, some improvements would make the proposed method applicable to the images taken in some different environments. When the images were taken under different lighting conditions, we can apply the proposed method to the images by adding the different lighting condition images for training the network and the regression model for estimating tiller numbers. When using the images taken with noisy backgrounds, we can apply the proposed method by using a plant detection method such as Amean et al., 2021 to delete the noisy background.

In future work, we will use other pretext tasks to learn the feature representations. The mechanisms of the pretext tasks remain obscure, and it is not known what kinds of pretext tasks are most effective for a given object task. Therefore, we will attempt to determine the most appropriate pretext task for the object task by trial and error. We also plan to apply the proposed method to other grass plant family such as wheat and rice.

Additionally, we will apply the proposed method to other plant phenotyping tasks. The proposed method assumes that few labeled training data are available. This is typically true of plant phenotyping tasks because many appearance traits are measured manually. We expect that the proposed method will be helpful in automating the measurement of various traits.

## 5 Conclusion

This paper has proposed a DNN-based tiller estimation method that achieves improved performance compared with conventional methods. The proposed method uses two separate models for feature extraction: a pretrained VGG-16 model and a model produced by solving pretext tasks. We considered both SVR and LR to estimate the tiller numbers. Experimental results show that the pretrained model and the model based on pretext tasks allow the proposed method to outperform the conventional approach.

## Data availability statement

The original contributions presented in the study are included in the article/**Supplementary Material**. Further inquiries can be directed to the corresponding author.

## Author contributions

YU and MI contributed to the conception and design of the study. RK performed the statistical analysis. KK prepared the materials for the research. YU wrote the first draft of the manuscript. All authors contributed to the article and approved the submitted version.

## Footnotes

1 In computer vision and pattern recognition area, image features which are determined by reserachers are called “hand-crafterd features,” contrast to the deep-learning-based features, which are automatically determined by training.

2
https://doi.org/10.6084/m9.figshare.1272859.v12

3
https://plantcv.danforthcenter.org/pages/data-sets/2013/setaria_burnin2.html.

4
https://plantcv.readthedocs.io/en/stable/tutorials/vis_tutorial/

5
https://plantcv.danforthcenter.org/

6
https://scikit-learn.org/stable/
