# Supplementary material for: Tiller estimation method using deep neural networks
Source: Front Plant Sci. 2023 Jan 13;13:1016507. doi: 10.3389/fpls.2022.1016507 (PMC9880423; doi:10.3389/fpls.2022.1016507)
Supplement: Supplementary file 1 [file DataSheet_1.pdf]

Supplementary Material

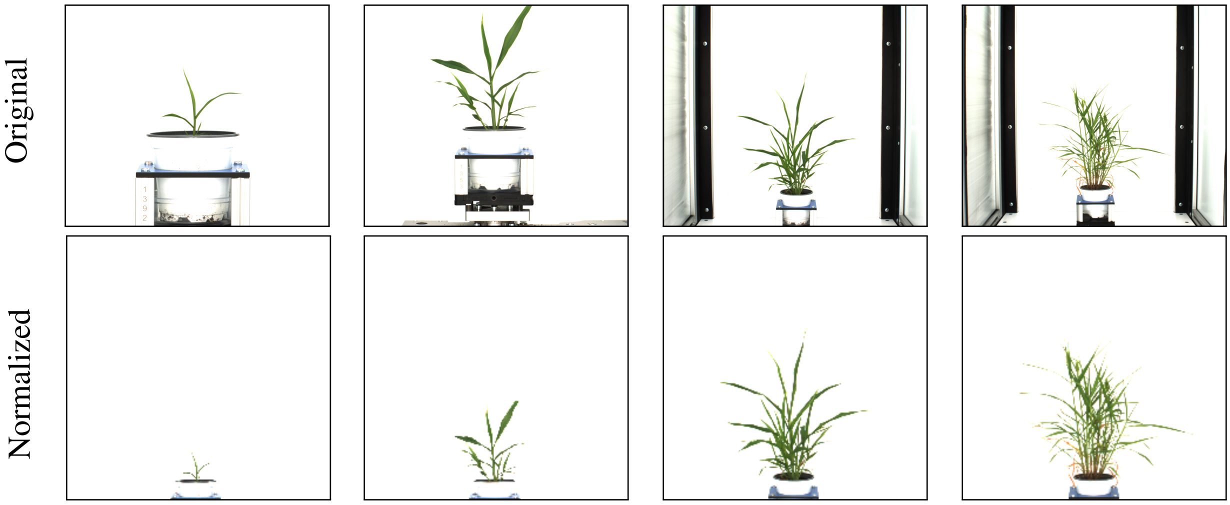

Figure S1. Examples of the images used for the experiments. The first row shows original images taken at different magnifications. The second row shows normalized images in which the pot is the same size.
